# Plant trait measurement in 3D for growth monitoring

**DOI:** 10.1186/s13007-022-00889-9

**Published:** 2022-05-03

**Authors:** Abhipray Paturkar, Gourab Sen Gupta, Donald Bailey

**Affiliations:** Department of Mechanical and Electrical Engineering, School of Food and Advanced Technology, Massey University, Palmerston North, New Zealand

**Keywords:** 3D modeling, Plant phenotyping, Plant growth monitoring, Structure-from-motion

## Abstract

**Background:**

There is a demand for non-destructive systems in plant phenotyping which could precisely measure plant traits for growth monitoring. In this study, the growth of chilli plants (*Capsicum annum* L.) was monitored in outdoor conditions. A non-destructive solution is proposed for growth monitoring in 3D using a single mobile phone camera based on a structure from motion algorithm. A method to measure leaf length and leaf width when the leaf is curled is also proposed. Various plant traits such as number of leaves, stem height, leaf length, and leaf width were measured from the reconstructed and segmented 3D models at different plant growth stages.

**Results:**

The accuracy of the proposed system is measured by comparing the values derived from the 3D plant model with manual measurements. The results demonstrate that the proposed system has potential to non-destructively monitor plant growth in outdoor conditions with high precision, when compared to the state-of-the-art systems.

**Conclusions:**

In conclusion, this study demonstrated that the methods proposed to calculate plant traits can monitor plant growth in outdoor conditions.

## Introduction

Agriculture is one of the important factors that humanity relies on. United Nations have included a goal in their 17 sustainable goals to promote sustainable agriculture to provide sufficient food for everyone with the aim to end hunger [1]. One of the goals is to improve crop production and plant breeding efficiency to successfully meet the growing food demands of more than nine billion people by 2050 [2]. Plant phenotyping provides vital information about crops which is helpful to farmers for their decision making process. Conventional phenotyping is manual, which is tedious, prone to errors, and labour intensive [3]. Plant-phenotyping is a set of protocols and techniques used to precisely calculate plant architecture, composition, and growth at different plant growth stages. Popular plant traits for growth monitoring include stem height, stem diameter, leaf area, leaf length, leaf width, number of leaves or fruits on the plant, and biomass [4].

Plant-phenotyping is an important area of research in plant breeding. It is implemented by a fusion of several techniques, such as spectroscopy, non-destructive imaging, and high performance computing [4]. Plant traits are measured at different scales, e.g. at the level of organs, plants, and canopies. These measurements can be performed using 2D or 3D imaging techniques. Some 3D imaging techniques, such as laser triangulation produce geometrically precise 3D plant models which enable accurate extraction of plant features. Some existing non-destructive phenotyping systems use 2D hyperspectral imaging [5, 6], or stereo imaging methods for calculating structural parameters of the plant [7, 8].

Image-based 2D methods can also help to extract plant traits. Systems often consist of a single camera mounted above the plant to produce a top view, occasionally combined with one or two more cameras to produce side views to calculate the leaf area or biomass of the plant [9]. However, calculation of biomass of a plant using 2D images has limited accuracy because these techniques depend on the position of the camera relative to the plant (since whole plant is not visible from a single 2D camera). Precise leaf measurements can be achieved if the camera inspects a view perpendicular to the leaf. Nonetheless, this cannot be guaranteed in practical set-ups, providing inaccurate leaf measurements.

### 2D systems for plant measurement

A semi-automatic phenotyping system presented in [10] uses 2D images to monitor plant rosette growth rate and expansion of size during its vegetative stages. This system constantly rotates the positions of the pots within the greenhouse environment to reduce the effect of micro-environmental conditions which is the strength of this system. However, since this system uses a single digital camera, it takes more time for data acquisition and processing. In the initial growth stage of the plant, 2D images can monitor the growth efficiently as the plant architecture is simple [11]. However, as the plant architecture gets more complex, 2D images cannot monitor the growth reliably. The system presented in [12] uses images taken from two different views (side and top view) for growth monitoring. During the growth monitoring period, different plant traits are measured, such as plant height and width. The measurements from this system were inaccurate as it was not able to handle occlusion efficiently, and struggled in the presence of shadows and reflections. As the plant size increased, the plant architecture complexity also increased making accurate measurement more difficult. Walter et al. [13] presented a system in which, the growth and leaf area was calculated using a camera mounted above the plants. The limited information about the plant is achieved as the camera provided only one view. A growth monitoring system is presented in [14], which helped to calculate traits of plants growing in different pots. This system captures images from two views (side and top view) to calculate water use and growth. Table 1 shows the advantages and disadvantages of these methods in detail.

**Table 1:** Summary of 2D systems reviewed in this section

Method	Viewpoint	Plant traits	Advantages	Disadvantages
Phenoscope [10]	Top	Drought stress	Reduces the effects of environmental variation	More amount of time between data acquisition and processing
R. Subramanian [11]	Side	Seedling root and root tip	Low-cost system and high image resolution	Limited to seedling-level monitoring
HTPheno [12]	Side and top	Plant height and width	Cost-effective	Inefficient occlusion handling, struggles in shadows and reflections
GROWSCREEN [13]	Top	Leaf area and root growth	Cost-effective	Limited plant information
GlyPh [14]	Top and side	Water use and growth	Low-cost	Struggles with complex plant architecture

Clearly, 2D image-based methods have limitations such as inability to handle occlusion, not providing sufficient information about plant traits (since the 2D camera systems do not cover all the plant views), plant measurements depend on the orientation of the camera and leaf. These limitations lead to inaccurate plant trait measurements. The list of various plant traits is provided in Table 2. To overcome these issues, 3D imaging techniques have been used and documented in the literature [15].

**Table 2:** Various 2D plant traits considered in the literature

Structural	Physiological	Temporal
Plant height plant width leaf length stem height leaf angle	Temperature content stress level of leaves water level drought carbohydrate content	Leaf elongation rate plant growth rate stem angle trajectory leaf curvature rate reproduction of organs

### 3D systems for plant measurement

Image-based 3D methods can be divided into active and passive techniques, with the major approaches being LiDAR [16], structured light [16–18], structure-from-motion [3, 15], and stereo vision [19–21]. The advantage of using active techniques like LiDAR is that the some of the LiDAR products are robust against sunlight, since the sunlight interference affect the laser light’s wavelength. Also the LiDAR is able to get depth information in dim light. However, LiDAR has several disadvantages, such as some products have poor resolution (depending on the application, one can use high-end LiDAR to give better resolution). In plant trait measurement, it is essential to have high resolution sensor to reconstruct the plant precisely. Another disadvantage is the warm-up time is required for stable measurement [16], it needs multiple captures for occlusion handling, struggles with presence of dust, and the sensor is costly [3, 16]. Structured light systems have struggled to perform well in outdoor conditions due to insufficient contrast of the projected patterns within bright sunlight [22]. On the contrary, passive techniques can give high resolution, accurate measurement of plant traits, and low sensor cost. However, these require computationally more expensive processing e.g. GPU. These GPU are in constant demand and costly. Computation time also depends on the complexity of the plant architecture. In brief, passive approaches are mainly used in applications where accurate measurements, occlusion handling, and obtaining high resolution 3D models are important [15].

The popular passive approaches for 3D modeling are structure-from-motion (SfM), stereo vision, and shape from texture/silhouette/focus/shading [23–28]. Stereo vision system is the most widely used technique in the literature for obtaining 3D information. It provides high resolution depth data from two different views; however, it is restricted due to the texture of plants (it requires texture on the plants) and has a relatively high computation time. Shape-from texture/silhouette/focus/shading uses a single camera to capture images, making it simpler to set up than stereo vision but results in self-occlusion. SfM extends stereo imaging to construct a 3D model from a large number of input images, reducing problems related to self-occlusion, and correspondence. Nonetheless, SfM has several disadvantages, such as a large computation time and the quality of the 3D model can depend on the number of input images and the image quality. Table 3 shows the computation time taken based on number of images used for 3D reconstruction.

**Table 3:** Computation time for SfM based on number of images used for 3D reconstruction

Plant	Number of input images	Computation time (min)
Chilli	90	7.5
	78	6.4
	65	6
	50	5.1
	35	4.5
	25	3

In conclusion, every sensor and technique has its merits and demerits and their accuracy may vary. One should choose the sensors and techniques depending on the budget and requirements. In this study, we will be using SfM because of its cost-effective nature.

### Structure-from-motion based studies

In recent years, computer vision researchers have predominantly used SfM for generating high quality 3D models. SfM is implemented on a large collection of overlapped images to get sparse and dense point clouds and can be applied to a range of scales such as seedlings, plants, and trees. The potential of SfM was examined by Snavley et al. [29] who used Bundler software to reconstruct an object in 3D using hundreds of overlapping images acquired by a single camera. SfM detects and matches identical features in images acquired from different views. Distinctive features in each image are detected using a scale invariant feature transform (SIFT) [30]. This method has performed well in outdoor conditions.

Recently, many studies have been conducted on 3D plant reconstruction. Golbach et al. [31] used a shape-from-silhouette for 3D reconstruction of plants. This study considered the plant traits, such as leaf length, leaf width, stem height, and leaf area. However, this study has several limitations, firstly, the system can not precisely measure leaf length and width of the leaf is curled. Secondly, this study considered only seedlings for measuring plant traits which makes the 3D reconstruction and measurement process easier as the plant architecture is simple.

Yu et al. [32] used SfM for the reconstruction of a sweet potato plant by capturing images from different views. In this study, various plant features are considered for growth monitoring, such as leaf area, plant height, number of leaves on the plant, and leaf area index (LAI). High correlation was achieved between ground truth measurements and extracted measurements from the 3D model. However, this study did not consider other important plant traits, like leaf length and leaf width which are also important for plant growth monitoring.

Jay et al. [33] used SfM to reconstruct a crop row using a single camera mounted above the crop row having only one view (top view). This approach provided limited information about the crop, such as number of leaves, leaf length, and leaf width. Santos et al. [34] used SfM and used a computer-vision based image acquisition approach by moving a single camera over the crop to get overlapping images from different views to solve the occlusion problem. This study achieved good correlation between measured and ground truth values of the plant by reducing overall average percentage error. However, there is a need to consider more plant traits like leaf length and leaf width for growth monitoring in this study to make the system reliable [31]. Rose et al. [26] also used SfM for 3D plant modeling and extracted plant features of a 3 week old tomato plant, such as plant height, convex hull of the plant and leaf area. Overall this study reported good correlation between ground truth values and measured values. Paturkar et al. [35] used smartphone’s camera to capture the plant images and then used SfM for 3D modeling of the plants. Various plant traits were measured in this study including plant height, number of leaves, and leaf area index. However, this study did not consider measuring the stem height, leaf length, and leaf width when it is curled. This study demonstrated that it is possible to achieve good 3D modeling results using a smartphone camera.

All these studies used SfM for 3D modeling and used standard methods for plant trait calculation. In these studies, the primary aim was to extract the plant’s trait measurement accurately. However, some techniques have considered seedlings to work with which makes it easier to measure the traits. Also, some methods struggled to measure plant trait accurately when the plants are at a more advanced growth stage. In addition, even though some studies considered plants at an advanced growth stage, they did not measure the plant trait when the leaf and stem is curled. This is a very common problem in this area and none of the studies tries to address it. Therefore, there is need to consider additional features to monitor plant growth such as, leaf length and width which have rarely been considered in the literature. These features are important as the photosynthesis process is dependant on leaves, making leaf dimensions important traits in growth monitoring. Paturkar et al. [36] presented the range of traits one can consider based on the need. Such measurements need to be robust even if the leaf is not flat (which is common). In this study, we will consider these plant traits (leaf length and width) along with other traits, such as number of leaves, and stem height. We will also look at leaf trait measurements when the leaf is curled.

We propose a 3D plant trait measurement system that addresses these challenges. The feasibility of the proposed method to measure leaf length, leaf width, leaf area, stem height, and number of leaves was validated by evaluating the reliability and accuracy of measuring these traits at different growth periods of the plant and in outdoor conditions. The overall aim is to extract accurate plant traits from the 3D model. Our contributions include:

1. Considering additional plant traits for growth monitoring, such as leaf length and width along with number of leaves, and stem height.
2. Investigating a novel approach to measure leaf length by calculating the distance between apex (tip) of the leaf through considering additional points in the middle region of the leaf to the stipule (point where leaf attaches to the stem). This way we can measure leaf length accurately even if the leaf is curled.
3. Investigating a novel approach to measure leaf width by measuring the widest part of leaf. The widest part is perpendicular to the leaf axis from apex to stipule.
4. Leaf trait measurements when the leaf is curled.

The rest of the paper is organised as follows: methods used in this study are explained in “Methods” section, with ground truth measurement presented in “Ground truth measurements” section. Detailed results are described in “Results” section. “Comparison with the state-of-the-art” section compares the proposed system with state-of-the-art systems. We end the paper with brief discussion along with conclusion and future scope in “Discussion and conclusion” section.

## Methods

### Experimental set-up

**Fig. 1:**
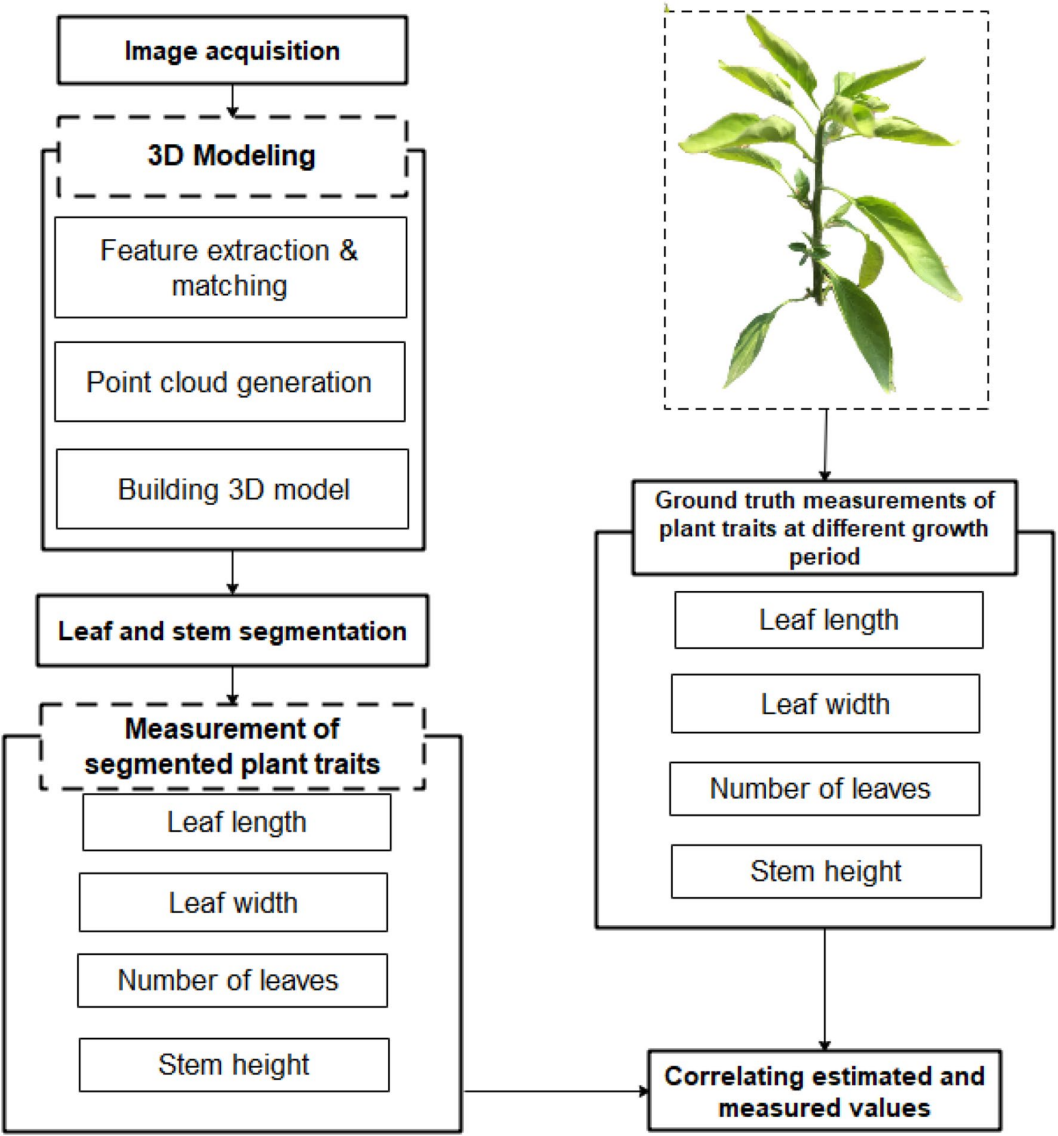
Flowchart of 3D plant reconstruction and plant trait measurements

We considered chilli plants (*Capsicum annum* L.) grown on commercial land in Palmerston North, New Zealand. We selected the chilli plant because it has constant demand and high value over the year. Chilli plant seedlings were transplanted in February 2020 and the growth of these seedlings was monitored twice a week from March 2020 till April 2020. We aimed at monitoring individual plants, so the plants were well-spaced so that other plants in the surrounding are did not interfere in the model. The plant traits measured include leaf length, leaf width, stem height, and number of leaves. Manual measurement for ground truth was obtained by counting the number of leaves, and measuring leaf length, leaf width, and stem height using a ruler scale. The detailed flow chart for 3D plant reconstruction and plant trait measurement is shown in Fig. 1.

**Fig. 2:**
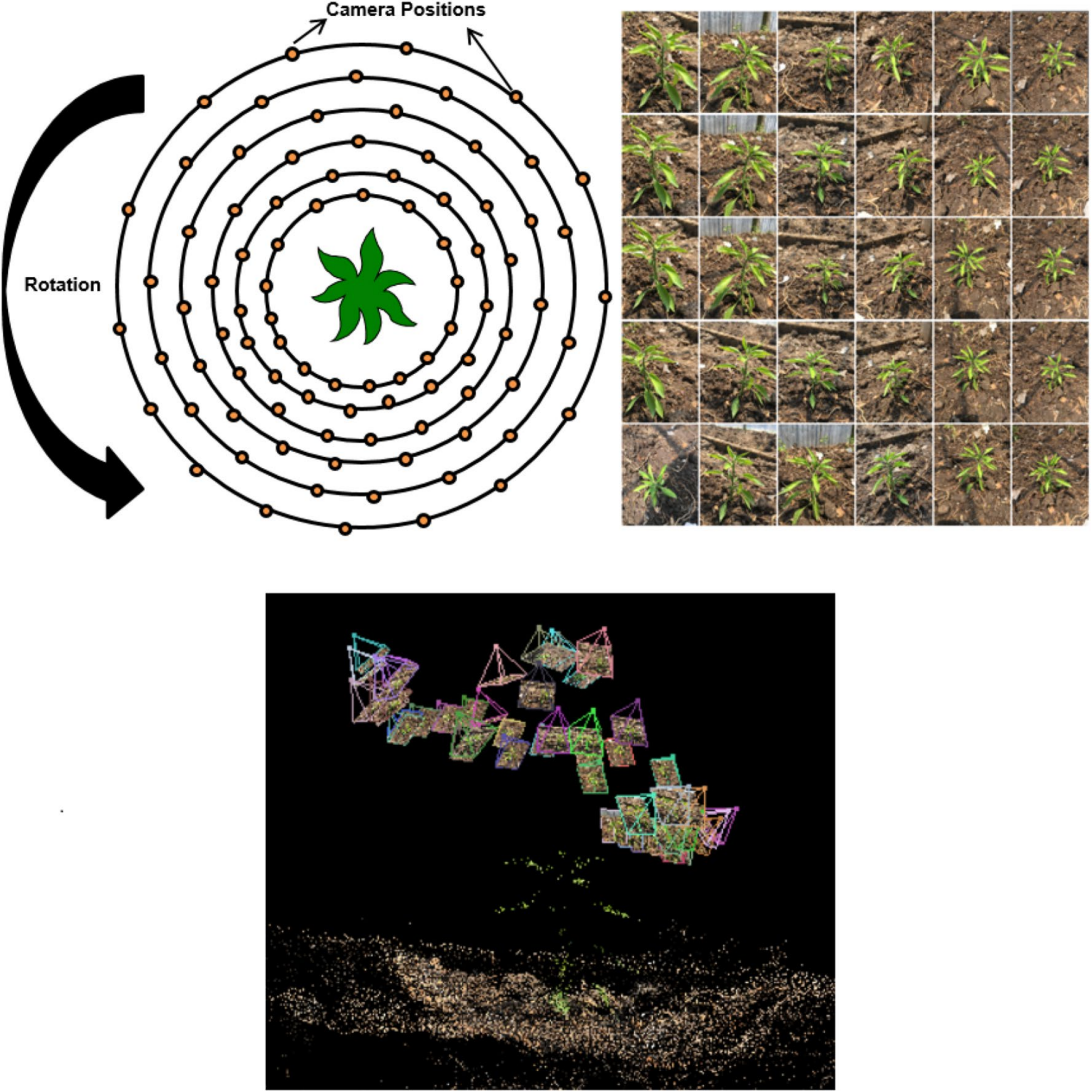
Image acquisition scheme, sample images acquired, and the camera angles (triangles) toward the plant

### 3D image acquisition

Images of the plant were captured using a mobile phone’s main camera (Apple iPhone 6s Plus with 12MP, f/2.2, and 29 mm focal length). Images were captured sequentially with the camera following an approximately circular path around the plant axis. Six rounds were captured from different distances and heights. Around 15 images were captured at approximately 25$^{\circ }$ intervals in each round. The distance of the camera from the plant was kept between 10 and 40 cm. This acquisition process produced up to 90 images with 95% overlap between images. The actual number varied depending on the growth stage and corresponding complexity of the plant architecture. Table 4 shows the number of input images with the growth of the plant. At each location, the camera was oriented in such a way that the complete plant was in the field of view. No camera calibration is required as the SfM algorithm determines both camera intrinsic and extrinsic parameters which is an important advantage of using this technique. The image acquisition scheme, the camera angle toward the plant (triangles in the image are the camera perspectives), and a sample of images acquired from various locations (views) are shown in Fig. 2.

**Table 4:** Comparison of number of input images with the growth stage

Date of measurement	Number of input images
15/3/2020	18
19/3/2020	27
22/3/2020	35
26/3/2020	48
29/3/2020	60
2/4/2020	72
5/4/2020	78
10/4/2020	90

### 3D modeling of the plant

SfM is a widely used technique for 3D reconstruction [15, 37–41]. As shown in Fig. 1, 3D modeling has 3 main steps.

First, distinctive keypoints on the plant need to be found in each image. It is the matching of these keypoints from one view to another that enables a 3D model to be constructed. The scale-invariant feature transform (SIFT) [30] is used for this because the resulting keypoints are invariant to image scaling, rotation, and translation. The important steps in SIFT are as follows:

1. Scale-space extrema detection: The primary step of SIFT searches over all image locations and scales using a difference-of-Gaussian function to detect promising keypoints that are orientation and scale invariant.
2. Localisation of keypoints: Keypoints are filtered to remove those with poor stability. Stability is a measure of the sensitivity of keypoints to changes in position and scale.
3. Orientation assignment to the keypoints: The orientation of every keypoint is determined from local image gradient directions. These are calculated based on the detected scale to give scale invariance.
4. Keypoint descriptor: The pattern of local image gradients at the chosen scale in the area around each keypoint are used to form a (hopefully unique) descriptor. Gradients provide invariance to changes in illumination and are relatively insensitive to shape distortion.
5. Keypoint matching: These keypoints are matched between pairs of images of the chilli plant acquired from various angles and views. Bundle adjustment is used to form a sparse 3D point cloud of the plant and retrieve camera positions and intrinsic parameters simultaneously.

Once features from different views are extracted and matched, a sparse 3D point cloud is produced. This sparse model is filtered to remove outliers, and other artefacts (caused by keypoint mismatching) and irrelevant reconstructed topography. Subsequently, the estimated camera positions, orientations, and parameters are used to generate a dense 3D point cloud using a form of stereo matching. Cross-correlation is used to match a pixel in one image with the corresponding pixel in the next image on the epipolar line [42]. This method is repeated for each and every overlapped pair of images.

**Fig. 3:**
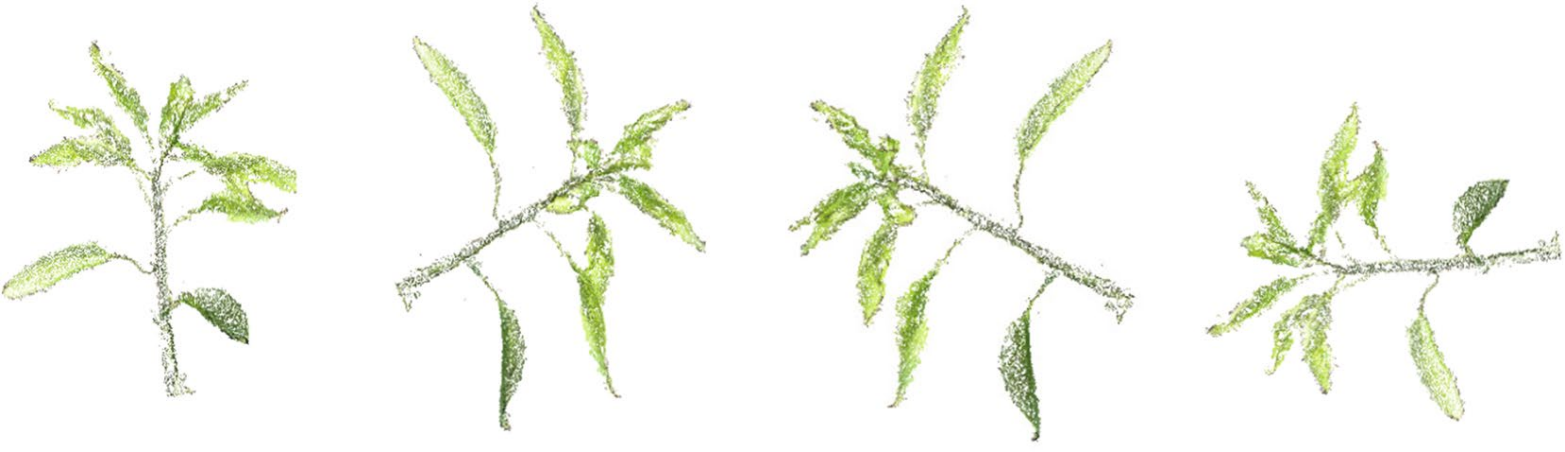
Four different views of a 3D model of a chilli plant

The generated sparse and dense point cloud is processed (smoothed and cleaned) using filtering and remeshing tools in *Meshlab* [43]. Figure 3 shows four different 3D reconstructed views of a month old chilli plant. The plant architecture was quite complex at this growth stage and still the chilli plant is reconstructed accurately. The stem and leaves are clearly visible. The same 3D model is used later in this paper to illustrate segmentation and trait extraction.

### Discussion of 3D modeling of the plant

We intentionally selected SfM for the reconstruction process because of its high flexibility. The number of images and its viewpoints do not need any calibration which makes the process simpler. A large number of images can be processed without preprocessing. However, SfM does require significant computation time. The computation time and memory depend on the number of images used for reconstruction. This varies with the growth stage.

Furthermore, the quality of the reconstructed plant 3D model also depends on the number of images being used for reconstruction. Fewer images will provide a less accurate 3D model. In contrast, increasing the number of images will result in a more accurate 3D model but processing redundant information from overlapped images will increase computation time. Therefore, it is important to have an appropriate number of input images to balance between computation time and the quality of the 3D model. The 3D modeling method explained in “3D modeling of the plant” section will be repeated on different subsets of randomly selected images for a particular set of input images. The size of the subset for this chilli plant is varied from 25 images through to 78 images. For each subset size, the experiment is repeated five times, selecting a different random subset. The quality of the reconstructed 3D model was determined by comparing the features extracted from the model with ground truth data (manually measured values of the actual plant). Plant features, such as stem height and number of leaves were extracted. By exploring the correlation of extracted features with ground truth values, the number of images required to give an accurate reconstruction of chilli plant was determined. The number of input images for 3D reconstruction varied in this study as the plant was growing. Fewer images reconstructed a poor 3D model based on the correlation between ground truth and extracted features. In contrast, more images reconstructed a relatively accurate 3D model. Please refer to [44] for detailed explanation. In addition, SfM has helped to tackle the issues reported in the literature for other reconstruction methods, such as self occlusion and the correspondence problem.

### Plant trait segmentation

In our previous work, we developed a plant trait segmentation algorithm [45] (please refer to this article for more details) which uses Euclidean distance for segmentation of the point cloud. Also, this algorithm does not require prior knowledge about the plant architecture. The point cloud is first pre-processed to the remove background and outliers, and the point cloud is down-sampled if it is required. Once the point cloud is pre-processed, it is then used for further analysis, and segmentation. A brief outline of the segmentation algorithm is presented here.

In plant point clouds, the cluster size varies from region to region. For instance, the leaf cluster may have different point cloud size to that of the stem cluster.

Let *P* be the set of points in the point cloud being segmented with a radius threshold $r_{th}$. Two points, $p_i, p_2 \, \epsilon \, P$ are adjacent if:

1 $$min \mid \mid p_i \, - \, p_t\mid \mid < r_{th}$$

Points $p_i \,$ and $\, p_j$ are in the same cluster $C_i$ if they are connected by a path of adjacent points. Consequently, points are in different clusters if there is not such a path, i.e.:

2 $$min \mid \mid p_i \, - \, p_j\mid \mid \ge r_{th}, \forall \, \, p_i \, \epsilon \, C_1, \, p_j \, \epsilon \, C_2$$

**Fig. 4:**
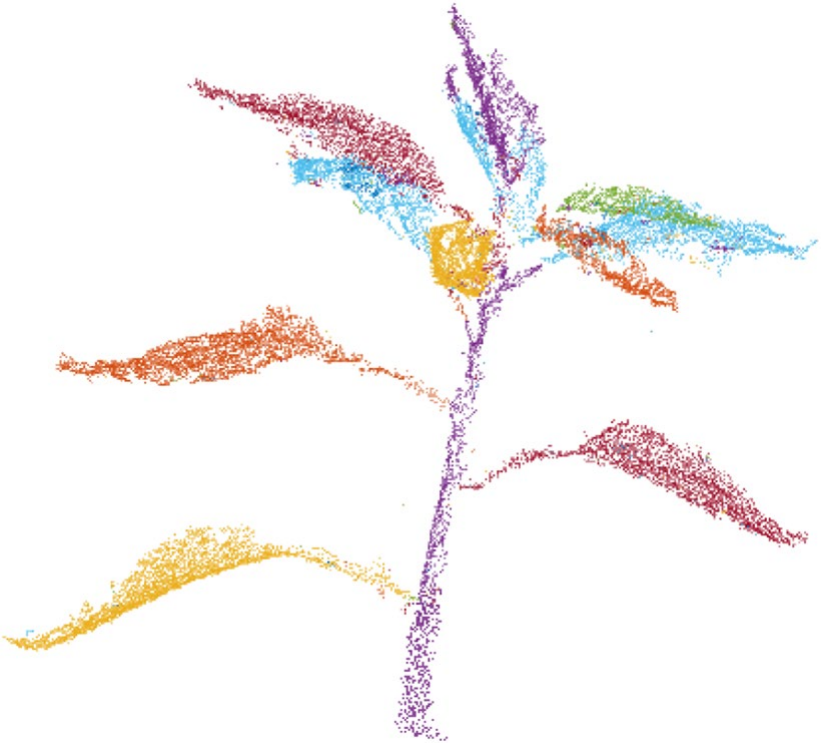
Plant trait segmentation

An appropriate value of $r_{th}$ can be found using an iterative search. This algorithm has performed well on different plant species and various plant architectures. Figure 4 shows the result of plant segmentation. It can be clearly seen that the algorithm segments the plant accurately.

**Fig. 5:**
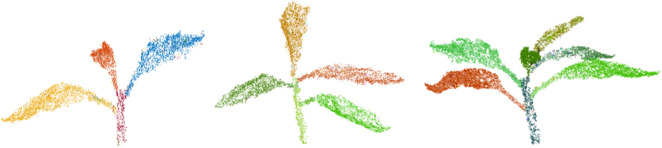
Additional segmentation results of the proposed method at different plant growth stages. Orientation of the 3D segmented model is kept in a way to visualise the segmentation results clearly and therefore, it is different at various growth stages

Figure 5 shows the segmentation results of the proposed method at different growth stages. This illustrates that proposed segmentation method has segmented plant’s stem and leaves efficiently and accurately at various growth stages of the plant. We repeated the same method at all the growth stages and achieved accurate segmentation results.

### Plant trait measurements

After leaf and stem segmentation, important plant traits for growth monitoring are measured: leaf length, leaf width, number of leaves, and stem height. These traits are important as the leaf size plays a vital role in the plant growth. In this study, we repeated these proposed measurements for all growth stages of the plant to validate its accuracy and reliability.

#### Leaf length

**Fig. 6:**
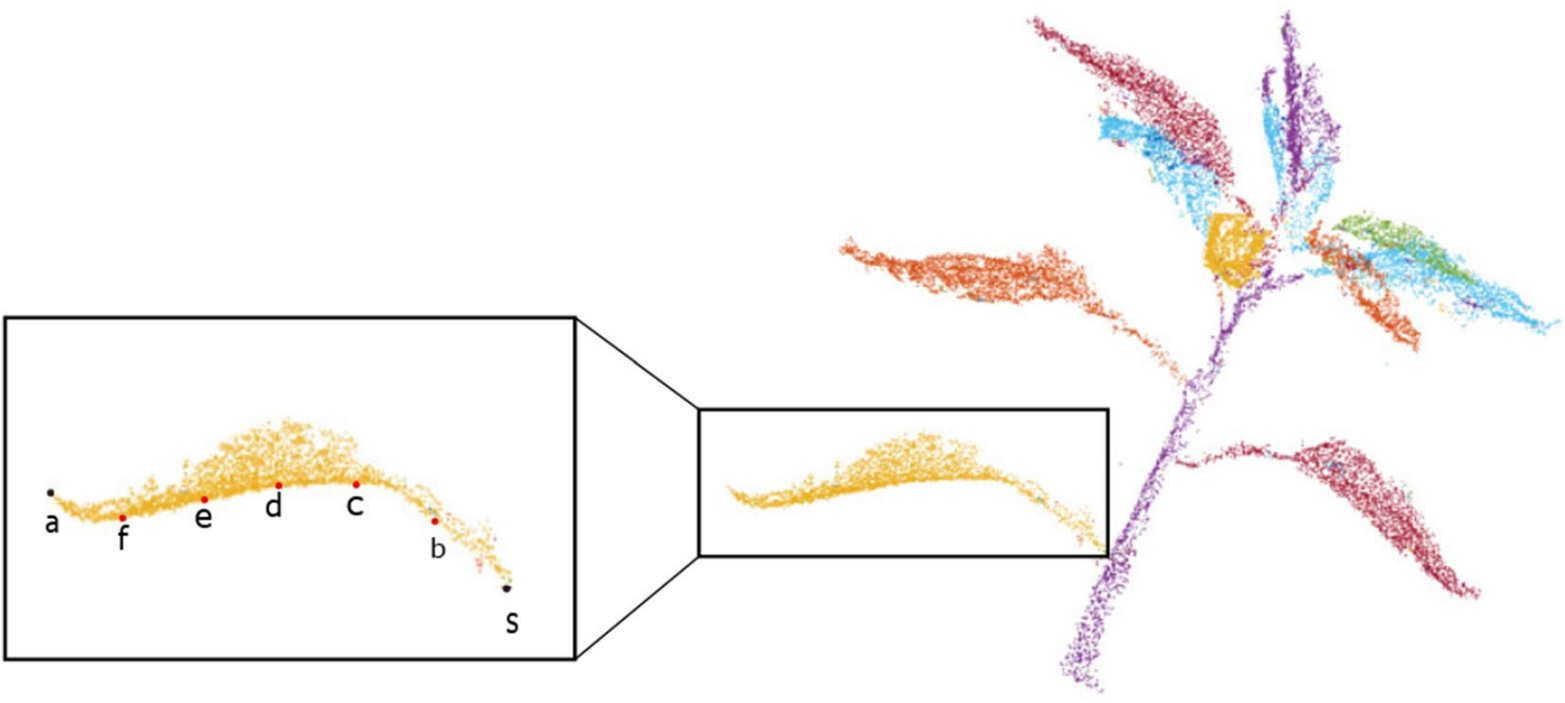
Leaf length measurement

Our definition of leaf length is the length along the midrib. The midrib is a strengthened vein along the middle of a leaf running from the apex (tip of the leaf) to the stipule (leaf’s connection to the stem). The midrib is detected using the method proposed in [46], which converts the leaf into its principal component analysis (PCA) coordinates to find the apex and the stipule. The segmentation method has precisely segmented each leaf (see Fig. 6). The apex of the leaf is defined as the point on the leaf which is furthest from the stipule. In contrast, stipule of the leaf is defined as the point on the leaf which furthest from the apex. These points are marked by black points *a* and *s* in Fig. 6.

In our study, we also measured leaves which are curled which makes the measurement challenging. If the leaf is straight then the Euclidean distance between apex and stipule will provide the leaf length. However, when the leaf is curled, this approximation will underestimate the true length. Therefore, to tackle this challenge, we consider additional points in between apex and stipule which will provide a close approximation of leaf length. We calculated the leaf length by considering 3 to 8 additional points between apex and stipule. However, the results from 5 to 8 points were identical and therefore, we selected 5 points to reduce computation time. These five points are marked by red points (*b-f*) in Fig. 6. The leaf length is measured using:

3 $$\begin{aligned} Leaf_{length}& ={\left\| \vec {s - b}\right\| + \left\| \vec {b - c}\right\| + \left\| \vec {c - d}\right\| }\\ & \quad + \left\| \vec {d - e}\right\| +\left\| \vec {e - f}\right\| + \left\| \vec {f - a}\right\| \end{aligned}$$

#### Leaf width

**Fig. 7:**
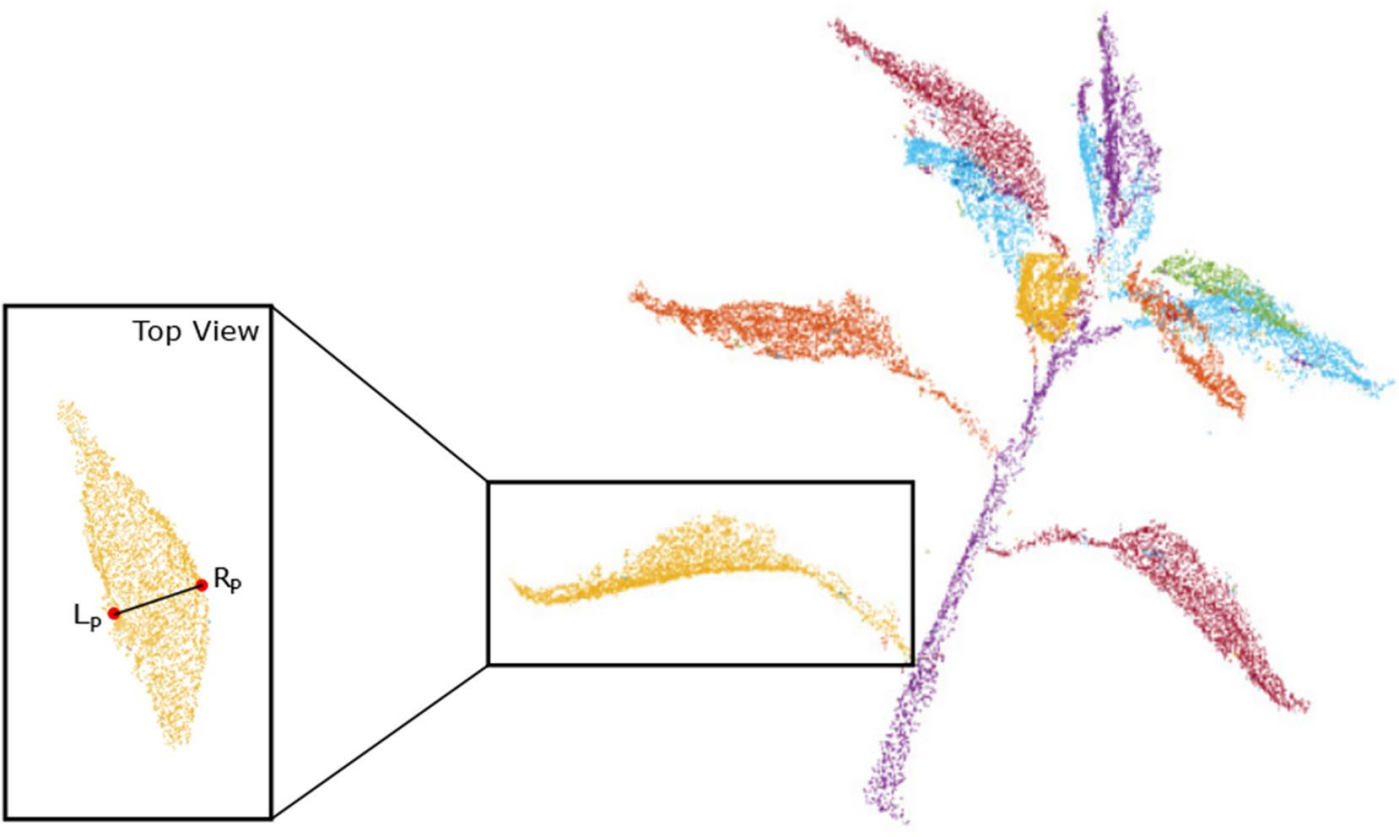
Leaf width measurement

To measure the leaf width, the widest region of the leaf which is perpendicular to the axis through the apex and stipule is found, marked by a black line in Fig. 7. The widest region on the leaf is determined by the part where the Euclidean distance between the two red dots is maximum. However, to find the location of these red dots is difficult. To do that, for all points on the leaf, the orthogonal projection on the line from the apex to stipule is measured. The distance between the apex and stipule is divided into 25 equidistant areas. The leaf points having projection in an area are selected, shown in red dots (Fig. 7). These red dots form a band across the leaf. The leftmost red point on the line, $L_{P}$ and rightmost point on the line, $R_{P}$ are used to approximate the width of that area. This step is repeated for all 25 areas; the leaf width is defined by the maximum of:

4 $$Leaf_{width} = max \left({{\left\| \vec {L_{P}-R_{P}}\right\| }}\right)$$

#### Stem height

**Fig. 8:**
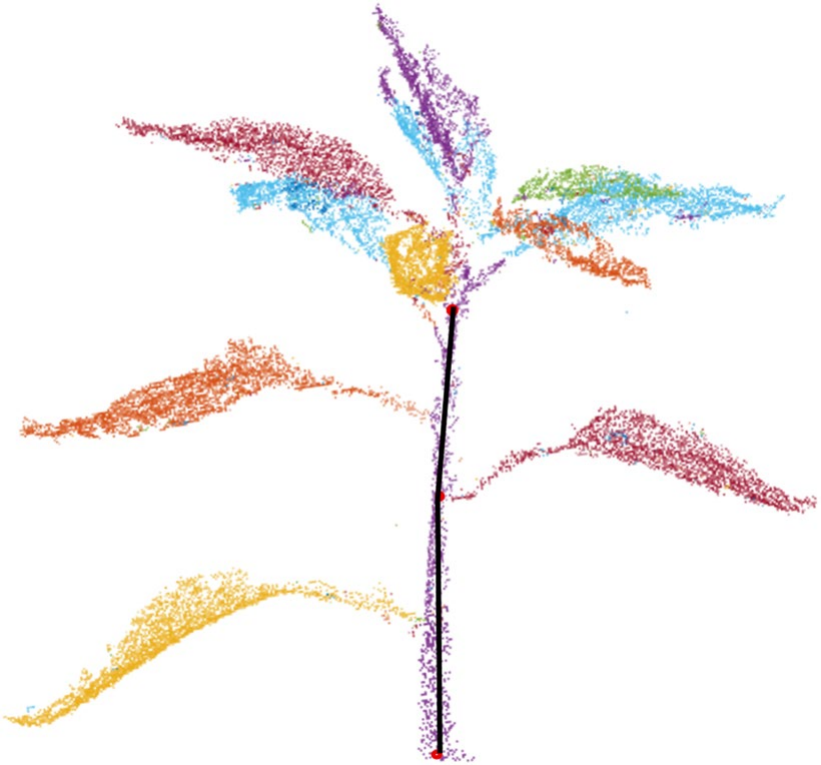
Stem height measurement

To measure stem height, we tried an approach used in [31] called mid-line tracking which scans the number of points on the stem. However, we analysed that in some cases at the initial growth stage of the plant when the stem is very short, the stem is not detected. In addition, in natural conditions, stem can be curved as well and hence a close approximation of true stem height is also needed. To tackle this problem and to measure stem height precisely, we selected three points on the plant stem. Figure 8 shows these three marked points, one point at the bottom of the stem, one at the middle and one at the point where the topmost leaf is connected. The stem height is measured by calculating the distance between these three points. The marked black points are selected by visual cues to measure stem height precisely.

#### Number of leaves

The number of leaves is another important trait of the plant for growth monitoring. Plant growth mainly depends on the leaves as they photosynthesise. During the segmentation process, we have already derived the stipule of the leaves. Therefore, we have the count of number of stipules in the model which ultimately provides the information about number of leaves.

## Ground truth measurements

In this study, to establish the ground truth, we measured different plant traits, such as leaf length, leaf width, number of leaves on the plant, and stem height. 8 sets of measurements were made (twice per week) during the period 15 March 2020 to 15 April 2020. The number of leaves varied over the growth period; Table 5 shows ground truth values of number of leaves and stem height.

**Table 5:** Ground truth measurements for number of leaves and stem height

Date of measurement	Number of leaves	Stem height (cm)
15/3/2020	3	5
19/3/2020	4	7
22/3/2020	6	10
26/3/2020	7	13
29/3/2020	8	15
2/4/2020	9	20
5/4/2020	10	23
10/4/2020	11	27

It is very easy and straight forward to count the number of leaves on the plant, simply by moving around the plant and carefully counting them. To measure leaf length, leaf width, and stem height, we used a conventional method of taking measurements with a ruler [32].

We define these measurements as the ground truth, although these measurements may not be accurate because of physical or human errors. For instance, some leaves are curled in such a way that it is difficult to measure accurately in the real world as the leaf may be damaged in the process of making it flat. However, careful measurements reduce these errors. In this study, we have considered these factors while calculating system accuracy.

## Results

In this section, the accuracy of the system is assessed based on regression between the ground truth values and measured values from the 3D segmented model at different growth stages. The correlation coefficient $(R^2)$, indicated the quality of the fit. In addition, the mean absolute percentage error (MAPE) of the measured values from our 3D model, $F _{t}$ and ground truth values, $A _{t}$ is used to assess the accuracy:

5 $$M= \frac{100\%}{n} \; \sum _{t=1}^{n} \; \left|\frac{A_t-F_t}{A_t} \right|$$

### Leaf length

**Fig. 9:**
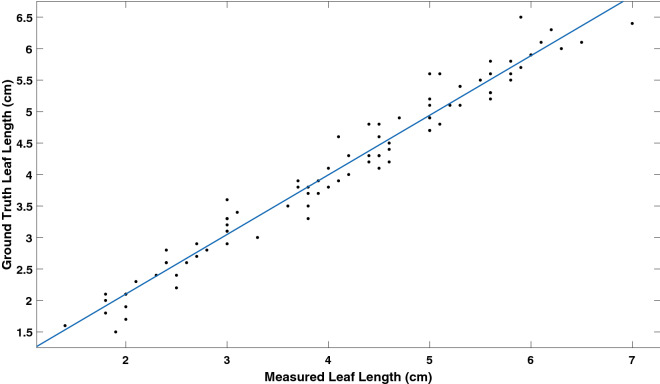
Correlation between ground truth and measured leaf length

The length of each of the leaves were measured individually over the period of 1 month. This gave a total 58 ground truth values and 58 measured values from the segmented 3D models for the leaf length. The leaf length varied from 1.6 cm to 6.5 cm over the period of the experiment. Figure 9 shows our proposed method performed well against the ground truth with a correlation coefficient of 0.97. The accuracy of the leaf length measurement method is determined by calculating RMSE which is 0.2 cm. The mean absolute percentage error is 5.8%.

### Leaf width

**Fig. 10:**
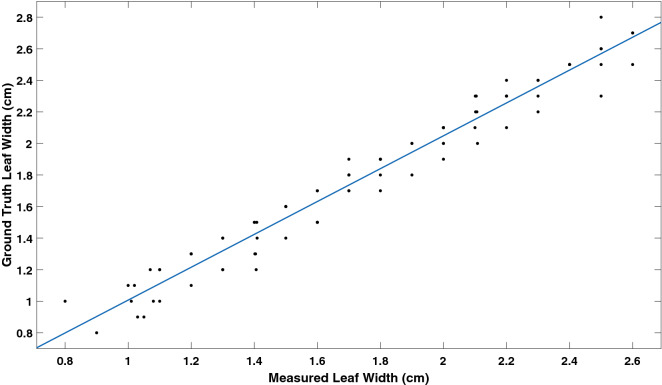
Correlation between ground truth and measured leaf width

Figure 10 shows high correlation between the measured values and ground truth values of leaf width with a correlation coefficient of 0.96 with RMSE of 0.11 cm. The mean absolute percentage error is 8% which is higher than that for leaf length because the width is one-third of the leaf length.

### Stem height

**Fig. 11:**
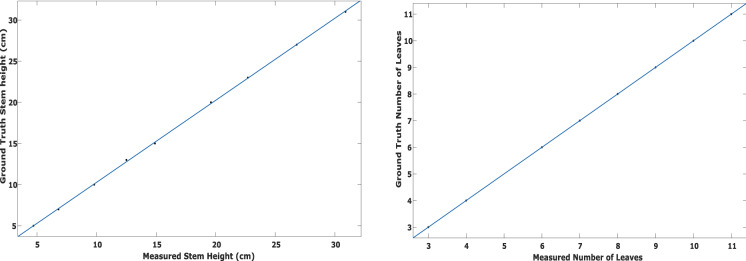
Correlation between ground truth and measured values for stem height (left) and number of leaves (right)

The stem height grew from 5 to 27 cm during the course of this experiment, with 8 ground truth values and 3D model values. The measurements from our proposed method showed high correlation with ground truth measurements as shown in Fig. 11 (left). The correlation coefficient for stem height is 0.99 with RMSE of 0.11 cm. The mean absolute percentage error is 2%.

### Number of leaves

The segmentation algorithm has performed extremely well to segment the leaves from the plant. Therefore, it is easy to count number of leaves. High correlation between the measured number of leaves and the ground truth is achieved with correlation coefficient of 1 as shown in Fig. 11 (right). The RMSE is zero as there is exact correspondence between measured and ground truth values.

## Comparison with the state-of-the-art

In this section, the proposed plant growth monitoring method is compared with different state-of-the-art systems. The detailed comparison is shown in Table 6.

**Table 6:** Comparative analysis of state-of-the-art systems

Method	Stem height		Leaf length		Leaf width		Number of leaves	
	RMSE (cm)	($R^2$)	RMSE (cm)	($R^2$)	RMSE (cm)	($R^2$)	RMSE	($R^2$)
Rose et al. [26]	0.14	0.96	*NA*	*NA*	*NA*	*NA*	*NA*	*NA*
Jay et al. [33]	1.1	0.99	*NA*	*NA*	*NA*	*NA*	*NA*	*NA*
Golbach et al. [31]	0.43	0.87	0.43	0.91	0.21	0.85	*NA*	*NA*
Hu et al. [47]	0.29	0.99	*NA*	*NA*	*NA*	*NA*	*NA*	*NA*
Yu et al. [32]	0.71	0.97	*NA*	*NA*	*NA*	*NA*	4.03	0.99
Paturkar et al. [35]	0.13	0.97	*NA*	*NA*	*NA*	*NA*	0.06	0.99
Our method	0.11	0.99	0.2	0.97	0.11	0.96	0	1

Golbach et al. [31] used shape-from-silhouette for 3D reconstruction of plants. This study considered similar plant traits, such as leaf length, leaf width, stem height, and leaf area. However, their system can not precisely measure leaf length and width if the leaf is curled. It also considered only seedlings for measuring plant traits, which makes the 3D reconstruction and measurement process easier as the plant architecture is simple. The proposed method has overcome these drawbacks and performs well even if the plant leaf is curled.

Zhang et al. [32], tested sweet potato in outdoor conditions and extracted different plant traits such as plant height, number of leaves, and leaf area from a 3D model. However, they did not consider important leaf traits such as length and width.

Hu et al. [47], used a Kinect sensor to monitor growth of a leafy vegetable and extracted plant height, leaf area and volume. The results were highly correlated with manual measurements but they did not test the system in outdoor conditions. The proposed approach achieves lower RMSE and good correlation between the measured and the ground truth values compared to Hu’s demonstrated system.

Rose et al. [26] proposed a photogrammetric method for precise measurement of growth parameters of 3 week old tomato plants in the greenhouse and demonstrated that all considered growth parameters are correlated with manual measurements. However, this study did not examine performance of the system in outdoor conditions. The proposed system achieved better correlation for plant height compared to their study.

Jay et al. [33] proposed an approach to extract structure parameters for five different plants, which are leaf area and plant height. These two parameters are highly correlated between calculated and manually measured values. Nonetheless, their approach did not deal with occluded leaves and did not have additional information to extract leaf length, leaf width, and number of leaves.

Paturkar et al. [35] which is a part of our previous work, measured stem height, number of leaf, and leaf area. However, this study did not address the issue to measure the curled stem. The proposed method demonstrated that even a mobile phone can be used for image capture to reconstruct the plants in 3D giving an $R^2$ > 0.96 for leaf length, leaf width and also, $R^2$ > 0.99 for stem height and number of leaves.

## Discussion and conclusion

In this paper, methods are proposed to measure plant traits for growth monitoring based on reconstruction of plants in 3D. Measured plant traits consist of leaf length, leaf width, number of leaves, and stem height. These are the basic, yet important, phenotypic features of the plant. We considered calculating leaf area in our previous study [35] and hence did not include this parameter as we wanted to cover possible parameters for plant trait measurement. The proposed method is accurate for plant trait measurements. Structure-from-motion is used for reconstruction of plants in 3D. The image acquisition process has been conducted in such a way that from every camera perspective, the plant is clearly visible. The image acquisition process was conducted manually with a user capturing images using a mobile phone camera without using a tripod or any other tools which made this process easy and adaptable. The time required for image acquisition is 2–3 min and this time increases with the growth of the plant as more images are required. The computation time per plant for post processing takes 10–12 min for generation of the point cloud, 7–12 min for removing outliers and filtering. The segmentation time has varied from 3.2 to 12.6 s and trait measurement time varied from 8.8 to 17.5 s throughout the growth stages of the plant.

The generated 3D model of the plant is then accurately segmented into leaves and stem parts. These segmented parts are then used to calculate plant traits. Leaf length and leaf width can be measured with an absolute mean percentage error of 5.8% and 8% respectively; error for stem height and number of leaves is 2% and 0% respectively.

The advantages of the proposed method include reduced user involvement for plant segmentation and precise plant trait measurements. The SfM algorithm estimates the intrinsic camera parameters automatically which removes the need for explicit camera calibration. In the initial growth stage the processing time was less but as the plant architecture gets more complex with the growth, the processing time increases. We believe that the processing time can be reduced with more efficient algorithms. Also, the results of stem and leaf segmentation show a limitation in processing high resolution point clouds. Also, the leaf and stem measurement techniques does not always find an optimal leaf and stem position. This can be improved by using skeletonization algorithm, which will provide a more accurate plant connections. This will help to improve plant segmentation as well as plant’s geometrical measurements. One of the major challenges in this study is to deal with heavy occlusion of the plant leaves, for instance, situations where the leaves are just connected or touching each other. Also, for plant phenotyping purpose, the accuracy should be increased in the future work. In addition, in terms of image acquisition sensor, there are various techniques available in the literature, but should choose the sensor based on the requirements and budget. Similarly, there are various plant traits available to consider for plant phenotyping in 3D, one must select these traits based on the plant.

The leaf length was measured precisely by considering 5 additional points in the middle region of the leaf from apex to stipule. This helped us to calculate leaf length accurately even when the leaf was curled with high correlation of 0.97 between ground truth and measured values. Similarly, leaf width is calculated by determining the widest part of the leaf with RMSE of 0.11 cm and correlation coefficient of 0.96. These methods helped to overcome the disadvantage associated with inaccurate measurement of curled leaves.

In conclusion, this study demonstrated that the methods proposed to calculate plant traits have the potential to monitor plant growth in outdoor conditions. Future work consists of applying this system on multiple plants to determine the robustness and reliability.

## Data Availability

The datasets during and/or analysed during the current study available from the corresponding author on reasonable request.
